# Chronic Obstructive Pulmonary Disease: The Role of Healthy and Unhealthy Dietary Patterns—A Comprehensive Review

**DOI:** 10.1002/fsn3.4519

**Published:** 2024-10-30

**Authors:** Mohammad Vahedi Fard, Kimia Mohammadhasani, Zahra Dehnavi, Zahra Khorasanchi

**Affiliations:** ^1^ Department of Nutrition, Food Sciences and Clinical Biochemistry, School of Medicine, Social Determinants of Health Research Center Gonabad University of Medical Sciences Gonabad Iran; ^2^ Department of Nutritional Sciences, Faculty of Medicine Mashhad University of Medical Sciences Mashhad Iran

**Keywords:** chronic obstructive pulmonary disease, diet, dietary patterns, nutrition, respiratory health

## Abstract

Chronic obstructive pulmonary disease (COPD) is a progressive and irreversible disease affecting many people worldwide. Recent evidence suggests that diet and lifestyle play a vital role in COPD progression. We aimed to provide a comprehensive review of the effect of healthy and unhealthy dietary patterns on preventing and treating COPD. For this reason, Scopus, EMBASE, Web of Science, and PubMed were searched. Based on our findings, it appears that adhering to a healthy dietary pattern rich in vegetables, legumes, fruit, nuts, and whole grains may have advantageous impacts on preventing and treating COPD while following an unhealthy dietary pattern rich in red and processed meat, saturated fats, sweets, and sugary drinks affect COPD negatively. Adhering to Mediterranean, dietary approaches to stop hypertension (DASH), Prudent, Ketogenic, and High‐protein diet may be related to a lower risk of COPD and improved pulmonary function. Conversely, Western and Ramadan Intermittent Fasting diets may elevate the prevalence of COPD. Proposing a nutritious diet that enhances pulmonary function could potentially be an effective approach to preventing and managing COPD. A comprehensive knowledge of the relationship between dietary factors and COPD can provide healthcare professionals with properly supported approaches to advise patients and empower individuals to make informed lifestyle decisions that are beneficial to improve their pulmonary health.

## Graphical abstract

COPD is a progressive disease affecting many people worldwide. Evidence suggests that environmental factors play a vital role in the development of COPD. Proposing a nutritious diet that enhances pulmonary function could potentially be an effective approach for preventing and managing COPD.

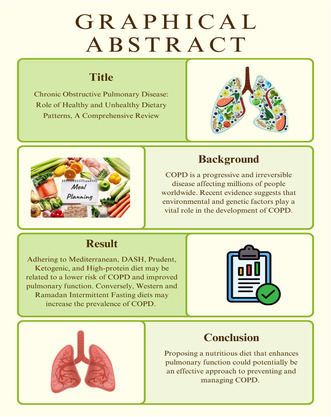

**Abbreviations**
AAT: alpha‐1 antitrypsin
COPD: chronic obstructive pulmonary disease
CRP: C‐reactive protein
DASH: dietary approaches to stop hypertension
ECOPD: exacerbations of COPD
FEV1: forced expiratory volume in one second
FVC: forced vital capacity
Hb: hemoglobin
hPDI: healthful Plant‐based Diet Index
Ht: hematocrit
IL: interleukin
low‐carb: low‐carbohydrate
MD: Mediterranean diet
ND: Nordic diet
NF‐κB: nuclear factor‐κB
NLRP3: nucleotide‐binding oligomerization domain‐like receptor 3
PaCO_2_
: arterial partial pressure of carbon dioxide
PaO_2_
: arterial partial pressure of oxygen
PUFA: polyunsaturated fatty acid
RBC: red blood cells
RIF: Ramadan intermittent fasting
ROS: reactive oxygen species
RQ: respiratory quotient
SCFA: short‐chain fatty acid
SERPINA1: the alpha‐1 antitrypsin gene
SFA: saturated fatty acid
SP‐D: surfactant protein D
TNF‐α: tumor necrosis factor‐α
WBC: white blood cells
WHO: World Health Organization

## 1 Introduction

Chronic obstructive pulmonary disease (COPD) is a systemic disease that includes a group of lung functional disorders, such as chronic bronchitis, emphysema, and small airway obstructions (Barnes, Shapiro, and Pauwels 2003; Kim 2022). COPD is a progressive and irreversible disease and causes a decrease in lung function (Buckner et al. 2021). In 2017, there were a total of 544 million cases of chronic respiratory disease worldwide, with 55.1% affecting men and 54.8% affecting women (Soriano et al. 2020). Based on the report from the World Health Organization (WHO), COPD ranks as the third leading cause of death globally, with nearly 90% of fatalities occurring in individuals under 70 years of age residing in low‐ and middle‐income countries (World Health Organization 2023b). It imposes substantial social, economic, and psychological burdens on the patient and society (Brakema et al. 2019; Rzadkiewicz, Bråtas, and Espnes 2016).

Studies mentioned that dietary habits and lifestyle interact with the pathogenesis of COPD as well as genetic and environmental factors (Agustí et al. 2022; Scoditti et al. 2019). Dietary habits are associated with body inflammatory status, nutritional deficiencies, weight management, and energy levels which directly affect lung health and COPD (Hanson et al. 2014; Heefner et al. 2024). The environmental risk factors are cigarette smoking, exposure to dust, fumes, chemicals, and air pollution (De Matteis et al. 2019; Venkatesan 2023; Yang, Jenkins, and Salvi 2022). Genetic factors also increase the risk of developing COPD and play an essential role in determining the likelihood of developing the condition (Stoller and Aboussouan 2005). Other risk factors for COPD include events during the fetal period, such as prematurity, poor growth in utero, and respiratory infections in childhood (World Health Organization 2023a).

COPD patients have symptoms, like chronic cough, dyspnea, chest tightness and wheezing, sputum production for 3 months or more in 2 successive years, anorexia, weight loss, and fatigue (Christensen et al. 2022; Kim and Criner 2013; Spruit et al. 2017; Venkatesan 2023). Spirometry is required in people with these symptoms and/or a history of exposure to COPD risk factors (Buist et al. 2007). The limited degree of airflow is mainly evaluated by the forced expiratory volume in 1 s (FEV_1_), forced vital capacity (FVC), and FEV_1_/FVC ratio (Vestbo et al. 2013). Also, the risk of some acute events increases in COPD patients, such as pulmonary embolism and decompensated heart failure (Vestbo et al. 2013). When dyspnea worsens and is accompanied by purulent sputum and cough, whereas there are no other symptoms in a COPD patient may be diagnosed as exacerbations of COPD (ECOPD) (Beghé et al. 2013). ECOPD will be assessed by heart rate, respiratory rate, C‐reactive protein (CRP), arterial partial pressure of oxygen (PaO_2_), and arterial partial pressure of carbon dioxide (PaCO_2_) (Venkatesan 2023).

Diet is a key factor in managing COPD. Based on WHO, a healthy dietary pattern is rich in vegetables, fruit, nuts, legumes, and whole grains. Also, < 30% of energy intake should be consumed from fats and unsaturated fats like olive oil, canola oil, and nuts are preferred. Furthermore, < 10% of energy intake should be consumed from free sugars, and < 5 g of salt should be consumed daily (World Health Organization 2023b). In contrast, an unhealthy diet is rich in processed foods, saturated and trans fats, and free sugar, whereas the intake of vegetables, fruit, and whole grains is insufficient (Jayedi et al. 2020). Mediterranean diet (MD), DASH diet, and Nordic diet (ND) are some examples of a healthy diet and the Western diet is an example of an unhealthy diet. Healthy diets rich in antioxidants can decrease the prevalence and severity of the disease by reducing inflammation and oxidative stress (Arslan et al. 2023; Scoditti et al. 2019). Additionally, diets that are rich in fiber content have a positive impact on the gut microbiome. Gut microbiota produces short‐chain fatty acids (SCFAs) by fermenting dietary fiber, improving gut barrier integrity, and regulating the immune system and inflammatory response (Ding et al. 2022; Kotlyarov 2022). Furthermore, dietary components may modulate the negative effects of genetic predisposition on the respiratory system by modulating genetic risk factors (Marín‐Hinojosa et al. 2021).

Some diet models are known as one of the most critical risk factors for COPD (Romieu 2005). Also, some dietary patterns play a pivotal role in both preventing and treating COPD (Kelly, Sacker, and Marmot 2003; Schünemann et al. 2002). The best approach to evaluate the advantages or disadvantages of nutrition for diseases, such as COPD is to assess a whole diet rather than individual foods or nutrients (Hu 2002; Schulze and Hoffmann 2006). Foods and nutrients are consumed together and may have antagonistic and synergistic interactions (Tapsell et al. 2016). Thus, using nutritional recommendations could be facilitated. There are not any studies that examine the effect of different healthy and unhealthy dietary patterns on COPD to show a comprehensive view in this context. Adjuvant treatments can be a cost‐effective option for COPD patients and also a preventive factor for individuals who are exposed to COPD risk factors. We aimed to investigate the role of healthy and unhealthy dietary patterns as adjuvant treatment in preventing and treating COPD.

## 2 Pathophysiology of COPD

**FIGURE 1:**
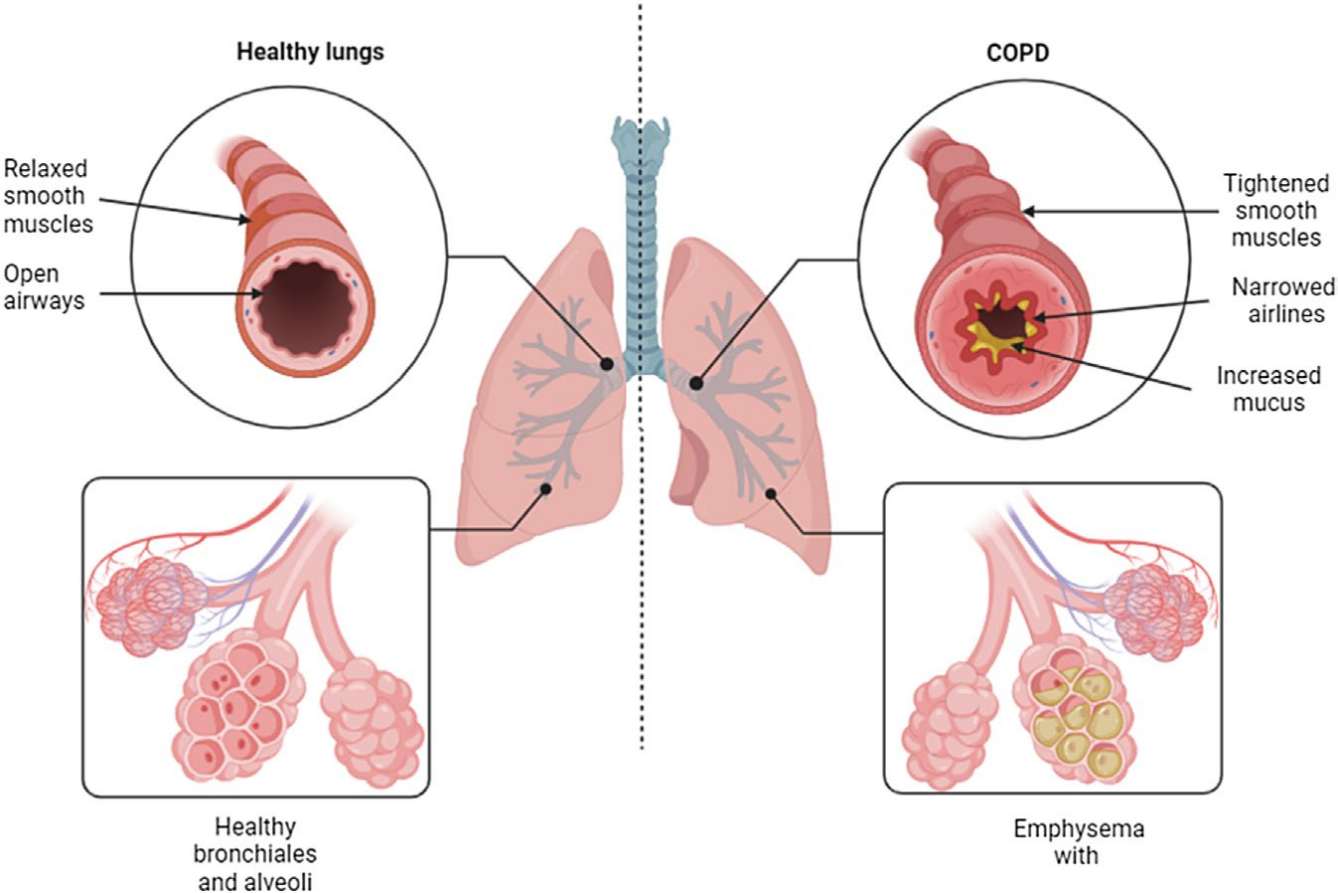
The contrast between healthy lungs and lungs affected by chronic obstructive pulmonary disease.

There are several pathogenic processes in the progression of COPD (Figure 1), such as the oxidative stress and inflammation response, pro‐catabolic status, gut microbial activity, apoptosis and cellular senescence, alteration of immune responses, cell proliferation, protease/antiprotease imbalance, and loss of elastic recoil by emphysematous destruction of parenchyma (Barnes, Shapiro, and Pauwels 2003). In all stages of COPD, oxidant and antioxidant abnormalities are common (Fischer, Voynow, and Ghio 2015). Inflammation of airways, epithelial cells, and immunology increases the amount of reactive oxygen species (ROS) in these patients, which worsens the condition of oxidative stress for patients (McGuinness and Sapey 2017). Also, inflammatory responses in the lung can be stimulated by the effect of oxidative stress on DNA, proteins, and lipids and destroy lung tissue (emphysema) (Di Stefano et al. 2002). In COPD, nuclear factor‐κB (NF‐κB) is an important nuclear factor in response to chronic inflammation that can regulate the expression of genes for pro‐inflammatory mediators, such as interleukin‐1 (IL‐1), IL‐6, IL‐8, monocyte chemoattractant protein‐1, and tumor necrosis factor‐α (TNF‐α) (Di Stefano et al. 2002). Also, chemotactic factors, such as macrophage inflammatory protein 1α and IL‐17A, can disrupt lung function by increasing oxidative stress and inflammation, causing emphysema, fibrosis of small airways, and stimulating lung irritation by inflammatory cells (Di Stefano et al. 2002; Schuliga 2015). Increased intestinal permeability has been reported in COPD patients. Changes in the gut microbiota can affect the severity of COPD (Sprooten et al. 2018). Bacterial products that enter the blood from the intestine are metabolized in the liver. The produced metabolite (trimethylamine N‐oxide) is related to increased long‐term mortality (Ottiger et al. 2018; Sprooten et al. 2018). Furthermore, genetic factors are known to be effective in causing COPD. One of the most relevant factors is the mutation in the alpha‐1 antitrypsin (AAT) gene (SERPINA1), which reduces a major inhibitor in the circulation of serine proteases called AAT (Stoller and Aboussouan 2005). The role of AAT is to protect the lung against neutrophil elastase and proteolytic enzymes (Stockley 2002). So, AAT structure changes cause polymerization and accumulation in liver cells and decrease the level of AAT (Lomas et al. 1992). Uninhibited neutrophil elastase due to AAT deficiency can lead to lung damage (Campbell et al. 1999; Liou and Campbell 1996).

## 3 Method

We carried out a non‐systematic review of the literature. A search of English‐language literature was conducted using Scopus, EMBASE, Web of Science, and PubMed. No restriction in time was applied. Articles were searched using the keywords “chronic obstructive pulmonary disease”, “chronic obstructive lung disease”, “COPD”, “diet”, “dietary pattern”, “Mediterranean diet”, “DASH diet”, “Prudent diet”, “Western diet”, “Intermittent Fasting”, “Ketogenic diet”, “low‐carbohydrate diet”, and “high‐protein diet”. We included articles that are relevant to the subject. Additionally, we included additional papers known to the authors.

## 4 Results

### 4.1 Mediterranean Diet

The Mediterranean diet (MD) is a healthy plant‐based diet. Currently, despite the reported evidence, this diet is considered for both preventing and treating chronic diseases, including obesity, metabolic syndrome, cardiovascular diseases, and high blood pressure (Cosentino et al. 2020; Lichtenstein et al. 2021; You 2015). MD is rich in the consumption of nuts, legumes, and fresh products, and also is especially rich in whole grains, fruit, vegetables, and extra virgin olive oil. MD is also characterized by moderate consumption of red wine during meals, fermented dairy products, poultry, fish, and seafood, and low intake of ready‐made meals, red meat, and sweetened beverages (Davis et al. 2015; Guasch‐Ferré and Willett 2021). MD contains antioxidants (β‐carotene, vitamins E and C), minerals, phytoestrogens, phenolic compounds (flavonoids), polyunsaturated fatty acids (PUFAs), and monounsaturated fatty acids (Davis et al. 2015).

**FIGURE 2:**
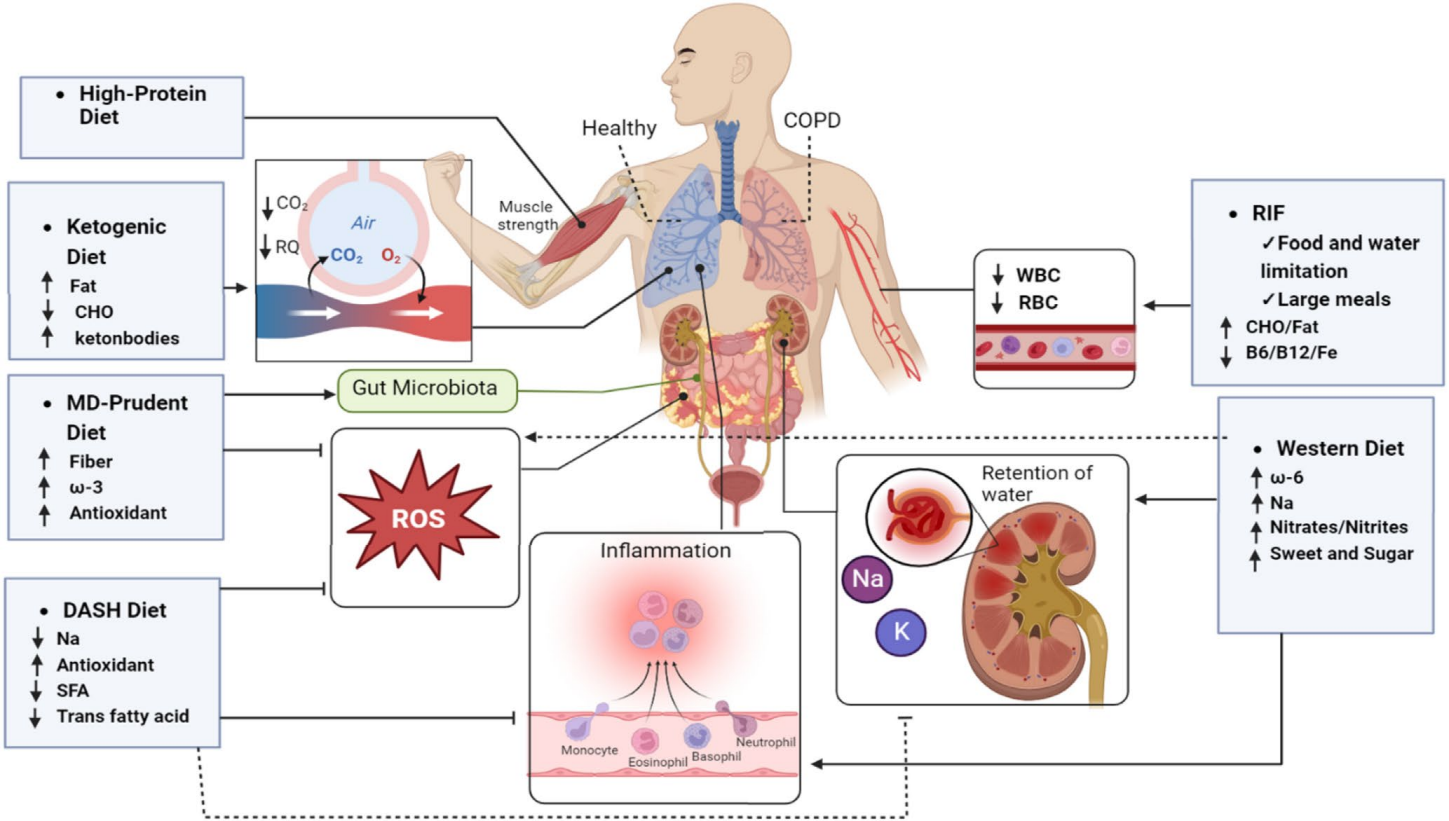
A comprehensive framework model illustrating the association between diets, dietary factors, lung function, and chronic obstructive pulmonary disease (COPD) development and progression. Mediterranean diet (MD), High‐protein, Ketogenic, Prudent, and dietary approaches to stop hypertension (DASH) diet is positively associated with COPD. Ramadan intermittent fasting (RIF) and Western diet are negatively associated with COPD.

Healthy dietary patterns like MD may be a protective factor for preventing and stopping COPD development (Figure 2 and Table 1). In a case–control study, Fischer et al. studied 370 individuals who visited with the diagnosis of COPD and compared it to 1432 controls. In the unadjusted model, people with high and moderate adherence to the modified MD score had a lower odds ratio in a dose–response manner to develop COPD than people with low adherence to the modified MD score. After adjustment, subjects with the highest and intermediate MD scores had a lower chance of developing COPD (Fischer et al. 2019). Analyzing 446 old COPD patients (more than 65 years) who were hospitalized because of the increased severity of COPD, Arslan et al., reported a significant negative correlation between high adherence to MD and frailty improvement in older adults with COPD. Also, following MD can improve dyspnea and the severity of COPD in these patients (Arslan, Bozkurt, and Bulut 2022). In their follow‐up study, they reported on the association between adherence to MD and fatigue as well as daily living activities. In older COPD patients, high adherence to the MD was found to attenuate fatigue and enhance independence in daily living activities (Arslan et al. 2023). Gutiérrez‐Carrasquilla et al. conducted a cross‐sectional study involving 3020 middle‐aged participants without any lung disease. Their aim was to examine the correlation between adherence to MD and spirometry values. FVC and FVE_1_ were significantly higher in participants with high adherence to the MD than in participants with low adherence to MD (Gutiérrez‐Carrasquilla et al. 2019). On the other hand, evaluating 121 COPD patients, Yazdanpanah et al. (2016) revealed that a high MD score was correlated with higher FVC and FEV_1_. Furthermore, better sleep quality in men with COPD who had higher MD scores was concluded by another study (Paknahad et al. 2020). Investigating 744 adults with acceptable spirometry values, Benslimane et al. reported that more adherence to MD with high intake of nuts, fruits, and cereals was negatively related to the risk of COPD. However, there was no significant correlation between COPD and the overall MD (Benslimane et al. 2022). In contrast, Wen et al., in their cross‐sectional study of 28,605 adults (2488 COPD patients and 25,607 non‐COPD subjects), evaluated the correlation between adherence to MD and COPD. The relationship between high adherence to MD and lower COPD prevalence was not significant (Wen et al. 2023). This study only analyzed the prevalence of COPD and did not contradict the improvement of COPD patients. Furthermore, some reasons can justify the non‐significance of the results. A group of participants may not be diagnosed correctly because the lung function was not measured.

**TABLE 1:** A review of studies that examined the association between dietary patterns and chronic obstructive pulmonary disease.

Authors	Type of study	Population	Association	Gene‐diet interaction results
**Mediterranean diet**				
Fischer et al. (2019)	Case–control	1802	Positive	Adherence to the Mediterranean diet reduced COPD development.
Arslan, Bozkurt, and Bulut (2022)	Cross‐sectional	446	Positive	High adherence to Mediterranean diet improves frailty in elderly people with COPD. Also, it improved the severity of the disease and dyspnea.
Arslan et al. (2023)	Cross‐sectional	526	Positive	High adherence to the Mediterranean diet decreased fatigue and increased independence in daily living activities in older COPD patients.
Gutiérrez‐Carrasquilla et al. (2019)	Cross‐sectional	3020	Positive	High adherence to the Mediterranean diet increased FVC and FVE_1_.
Yazdanpanah et al. (2016)	Cross‐sectional	121	Positive	High Mediterranean diet score was associated with higher FVC and FEV_1_.
Paknahad et al. (2020)	Cross‐sectional	121	Positive	Higher Mediterranean diet score was significantly associated with higher sleep quality in COPD patients.
Benslimane et al. (2022)	Cross‐sectional	744	Not significant	There was no significant correlation between COPD and the overall Mediterranean diet.
**DASH diet**				
Wen et al. (2023)	Cross‐sectional	28,605	Positive and no significant	Higher adherence to the DASH diet was significantly associated with lower risk of COPD. There was no significant association between high adherence to the Mediterranean diet and lower COPD prevalence.
Ardestani et al. (2017)	Case–control	84	Positive	Adherence to DASH dietary Pattern significantly reduced FEV_1_/FVC and cough in the control group.
**Prudent and Western diet**				
Varraso, Fung, Barr, et al. (2007)	Cohort	42,917	Positive and negative	Adherence to a Prudent diet reduced the risk of newly‐diagnosed COPD. High adherence to the Western diet increases the risk of newly diagnosed COPD.
Varraso, Fung, Hu, et al. (2007)	Cohort	72,043	Positive and negative	Adherence to Prudent diet reduced of risk of newly diagnosed COPD. High adherence to the Western diet increased the risk of newly diagnosed COPD.
Shaheen et al. (2010)	Cross‐sectional	2942	Positive	High adherence to a Prudent dietary pattern decreased prevalence of COPD in males. Also, it was significantly related to higher FVC in both sexes and FEV_1_ in males.
Steinemann et al. (2018)	Cohort	2178	Positive	Adherence to the Prudent dietary patterns was associated with high FEV_1_.
Varraso, Chiuva, et al. (2015)	Cohort	73,228 women and 47,026 men	Positive	Highest diet quality had a significant negative association with the risk of newly diagnosed COPD.
Dinparast et al. (2021)	Cross‐sectional	220	Positive	Healthy and mixed dietary patterns had a significant opposite association with depression of COPD.
Zheng et al. (2016)	Meta‐analysis	550,614	Positive and negative	High adherence to a healthy/prudent dietary pattern reduces COPD risk. High adherence to an unhealthy/Western diet increased the risk of COPD.
Varraso et al. (2023)	Cohort	73,592 women and 46,948 men	Positive	The highest healthful Plant‐based Diet Index score had a 46% reduction in the risk of developing COPD.
Sorli‐Aguilar et al. (2016)	Cross‐sectional	207	Negative	Adherence to a Westernized diet and impaired lung function (FEV_1_ < 80% and/or FVC < 80% and/or FEV_1_/FVC < 0.7) in women.
McKeever et al. (2010)	Cross‐sectional	12,648	Negative	Adherence to a traditional dietary pattern that is similar to a Western diet decreases FEV_1_ and increases the prevalence of COPD.
**Ramadan intermittent fasting diet**				
Rejeb et al. (2018)	Cross‐sectional	15	Negative/not significant	Ramadan intermittent fasting (RIF) reduces the WBC, RBC, hematocrit, and hemoglobin. Also, RIF had not significant effect on ESR and CRP indices. It significantly modified mid‐expiratory flow data too.
Zouari et al. (2018)	Cross‐sectional	16	Not significant	The spirometry data were not influenced by Ramadan intermittent fasting.
Mrad et al. (2019)	Case‐series	15	Negative	No significant association between RIF and oxidant stress biomarkers such as homocysteine, thiobarbituric acid reactive substances, and antioxidant stress biomarkers such as catalase, ceruloplasmin, superoxide dismutase, zinc, and albumin. Also, RIF had no significant effect on the number of high oxidant stress and low antioxidant stress status.
**Ketogenic and low‐carbohydrate diet**				
Malmir et al. (2021)	Case–control	336	Positive	Adherence to a low‐carbohydrate diet decreases the odds of COPD.
Cai et al. (2003)	Randomized clinical trial	60	Positive	Adherence to low‐carbohydrate diet reduced PaCO_2_, minute ventilation, oxygen consumption, carbon dioxide production, and RQ. Also, it increased PaO_2_ and FEV_1_ too.
Angelillo et al. (1985)	Randomized clinical trial	14	Positive	Adherence to low‐ and moderate‐carbohydrate diets, volume of carbon dioxide, respiratory quotient and arterial PaCO_2_ were significantly decreased. Also, FEV_1_ and FVC increased significantly.
Norwitz et al. (2021)	Case report	1	Positive	Adherence to the Ketogenic diet reduced granulocyte‐macrophage colony‐stimulating factor, TNF‐α, IL‐1β, IL‐6, IL‐8, and CRP. Also, FEV_1_ increased meaningfully.
**High‐protein diet**				
Yazdanpanah et al. (2010)	Cross‐sectional	63	Positive	The amount of protein intake had significant positive association with FVC and vital capacity.
Møgelberg et al. (2022)	Randomized clinical trial	13	Positive	Adherence to a high‐protein diet combined with physical activity improved peripheral muscle function.

MD is rich in whole grains, vegetables, fruit, and extra virgin olive oil (Davis et al. 2015). Hirayama et al. (2009) revealed that the consumption of fruit and vegetables by COPD patients was significantly lower than the control group. Also, Varraso, Chiuve, et al. (2015) indicated that high consumption of whole grains was related to a 30% lower risk of newly diagnosed COPD. These nutrients have high amounts of fiber. Multiple studies have shown a significant correlation between fiber intake and risk of COPD (Kaluza et al. 2018; Varraso, Willett, and Camargo 2010). For example, Szmidt et al. in a prospective cohort study of women, assessed the relationship between dietary fiber consumption and COPD. There was a significant correlation between the consumption of dietary fiber in the long term and a 30% reduction in COPD risk (Szmidt et al. 2020). There are many mechanisms for the function of dietary fiber in modulating inflammation. The intake of dietary fiber has been shown to decrease the concentration of inflammatory mediators, specifically CRP and IL‐6 (King, Egan, and Geesey 2003; Ma et al. 2006, 2008). Also, dietary fiber attenuates the risk of COPD through the intestine‐liver‐lung axis and modulates the innate immune system (Young, Hopkins, and Marsland 2016). This mechanism is focused on modulating the immune system through the effect of dietary fiber on the intestinal microbiome (Belkaid and Hand 2014). SCFAs are produced in the intestine by fermentation of fibers. They reduce the response of the immune system to pulmonary inflammation by activating protein G receptors on macrophages and neutrophils, inhibiting 3‐hydroxy‐3‐methylglutaryl‐coenzyme A reductase, histone deacetylase, and NF‐κB (Kau et al. 2011; Meier 2009; Trompette et al. 2014). This process also reduces inflammatory factors, such as CRP and IL‐6 (Marsland 2012; Maslowski et al. 2009; Sun et al. 2017). MD and its components, such as polyphenols and vitamins, affect epigenetic mechanisms like DNA methylation, non‐coding RNA, and histone modifications to modulate the expression of genes contributing to oxidative stress counterbalance, lung inflammation, proteinase imbalance, and apoptosis response (Marín‐Hinojosa et al. 2021).

Antioxidants provided by the components of MD may affect lung function and improve COPD (Enescu 2010; Orozco‐Levi et al. 2021). A 3‐year prospective study assessed the long‐term role between COPD patients who consumed higher antioxidant‐rich foods, such as fresh vegetables and fruit and patients who had an unrestricted diet. They indicated an annual increase in the percentage of FEV_1_ in patients who consumed high amounts of vegetables and fruit (Keranis et al. 2010). Consumption of high amounts of antioxidants decreases lung inflammation and modulates oxidative stress in COPD patients (Orozco‐Levi et al. 2021). Antioxidants like vitamins E, C, and A, and polyphenols play a protective role in this disease (Hirayama et al. 2009; Kirkham and Barnes 2013; Loukides, Bakakos, and Kostikas 2011). Vitamin E, A, and beta‐carotene, with their antioxidant property, reduces oxidative stress and lung inflammation by removing ROS (Barcia et al. 2012; Hirayama et al. 2009; Lippman 1989; Mousavi‐Shirazi‐Fard et al. 2021; Zhao et al. 2022). Vitamin C is an antioxidant that reduces dyspnea and wheezing in COPD patients by decreasing oxidative stress, increasing alveolar cells, restoring vascular endothelial growth factor, and increasing collagen synthesis (Koike et al. 2014). Also, this vitamin prevents the development of COPD (Keranis et al. 2010; Kodama et al. 2017). Polyphenols decrease levels of pro‐inflammatory mediators in COPD patients and reduce the severity (Fu et al. 2022). Resveratrol, a polyphenol that is rich in MD, is directly associated with lower inflammation, and oxidative stress and can be a candidate to counteract muscle and lung impairments characteristic of COPD (Beijers, Gosker, and Schols 2018). Olive oil, another unique component of MD, monounsaturated fatty acids, PUFA, pigments, tocopherols, polyphenols, phytosterols, and squalene that reduce oxidative stress and inflammation (Piroddi et al. 2017). Also, Olive oil in MD increases glutathione levels in COPD patients (de Batlle et al. 2010). Glutathione is a protective antioxidant against oxidative stress and modulates pro‐inflammatory processes in the lung (Rahman and MacNee 2000). COPD patients have lower blood levels of glutathione than others (Sotgia et al. 2020). Also, vegetable consumption decreases the levels of malondialdehyde (de Batlle et al. 2010). Malondialdehyde is produced by the intracellular peroxidation of PUFAs and is known as a marker of oxidative stress (Paliogiannis et al. 2018). Furthermore, vegetable intake increases FVC and FEV_1_ among COPD patients (Yazdanpanah et al. 2016).

MD recommends the consumption of nuts and fish as they are rich sources of omega‐3 fatty acids (Urquiaga et al. 2017). On the other hand, consumption of red and processed meat was reduced (Guasch‐Ferré and Willett 2021). Therefore, intake of omega‐6 fatty acids decreases (De Lorgeril and Salen 2012). Inflammation plays a vital role in the severity of COPD. The pro‐inflammatory properties of omega‐6 fatty acids and the anti‐inflammatory properties of omega‐3 fatty acids are important in inflammation (Patel et al. 2004). Adhering to MD reduces inflammation through balancing the omega‐6 fatty acids and omega‐3 fatty acids (Pella et al. 2003). Lower inflammation decreases fatigue in COPD patients (Mousavi‐Shirazi‐Fard et al. 2021). Moreover, a higher PUFAs/saturated fatty acids (SFAs) ratio after adhering to MD increases FVC and FEV_1_ in these patients (Yazdanpanah et al. 2016). Higher sleep quality is obtained by higher consumption of fruit, whole grains, fish, and PUFAs (Paknahad et al. 2020).

### 4.2 Nordic Diet

The Nordic diet (ND) is a health‐promoting diet based on the foods originating from the Nordic countries, including high consumption of fruits, legumes, whole grains, vegetables, fatty fish, low‐fat dairy, and canola oil and low consumption of sugar‐sweetened products (Ramezani‐Jolfaie, Mohammadi, and Salehi‐Abargouei 2019). The MD and ND are regarded as “plant‐based” dietary patterns that are similar in most of the components but the noteworthy point of difference is the oil utilized in them. ND is based on canola oil, whereas MD is based on olive oil (Lankinen, Uusitupa, and Schwab 2019). Canola oil has a lower concentration of phenolic compounds, but it contains a higher amount of phytosterols and tocopherols (Francisco et al. 2019). Canola oil also includes pigments such as chlorophylls and other trace elements, including ubiquinone (Coenzyme Q10), which plays a role in energy production and prevents peroxidative damage to membrane phospholipids as well as oxidation caused by free radicals (Martinez‐Gonzalez et al. 2009). ND components like MD, contain antioxidants that improve COPD through the mechanisms mentioned above. Furthermore, canola oil has a positive effect on COPD patients by its unique components and has been utilized in oral nutrition supplements for COPD patients (DeBellis and Fetterman Jr 2012). However, there are no studies that examine the effect of whole ND on COPD patients and needs primary investigations. ND emphasizes seasonal and locally sourced foods, which may not be available in all regions. Fresh products, whole grains, and fatty fish may not be available for some patients who live in different areas and make it difficult to adhere to ND.

### 4.3 DASH Diet

The Dietary Approaches to Stop Hypertension (DASH) diet is a healthy eating pattern that offers various beneficial effects on the body, including the ability to lower blood pressure (Shirani, Salehi‐Abargouei, and Azadbakht 2013; Wickman et al. 2021). It is considered by eating vegetables, whole grains, fruits, legumes, vegetable oils, seeds, nuts, fish, low‐fat dairy products, and poultry (Steinberg, Bennett, and Svetkey 2017; Wickman et al. 2021). Also, limiting consumption of salt, high‐fat dairy products, fatty meats, and sweets is necessary for this dietary pattern (Steinberg, Bennett, and Svetkey 2017; Wickman et al. 2021). The DASH diet contains high amounts of antioxidants, phytochemicals, fiber, and minerals (magnesium, calcium, and potassium) and low amounts of trans fatty acids and SFAs (Kerley 2018; Salehi‐Abargouei et al. 2013). Therefore, this diet can decrease oxidative stress and inflammation, especially in lung diseases (Soltani, Chitsazi, and Salehi‐Abargouei 2018).

Few studies have investigated the association between the DASH diet and COPD (Figure 2 and Table 1). Wen et al. in their study evaluated the relationship between DASH diet score and the risk of COPD. They showed that higher adherence to the DASH diet was significantly related to a lower risk of COPD (Wen et al. 2023). Analyzing 84 COPD patients and 80 non‐COPD participants, Ardestani et al. reported that adherence to the DASH diet was lower in COPD patients than control group. They found a significant reduction in the FEV_1_/FVC ratio in the control group that had higher adherence to the DASH diet, but other spirometry tests (FVC and FEV_1_) were not significant. Also, higher adherence to the DASH diet reduced cough significantly (Ardestani et al. 2017).

The DASH diet is a healthy diet that may improve COPD. It is rich in healthy components and reduced amounts of salt, fat, and sugar (Steinberg, Bennett, and Svetkey 2017; Wickman et al. 2021). One study indicated that COPD patients had lower consumption of dietary fiber, vegetables, whole grains, nuts, legumes, vitamin C, and vitamin E compared to healthy participants (Ardestani et al. 2017). Furthermore, a prospective cohort study indicated an inverse correlation between a higher intake of vegetables and fruit and COPD incidence (Kaluza et al. 2017). Another study revealed that a higher intake of dietary antioxidants was associated with higher FEV_1_ among COPD patients (Hong et al. 2018). As mentioned earlier, these components have positive effects on COPD and contribute to its improvement by modulating gut microbiome and epigenetics in the development of the disease. Also, a high intake of sodium leads to fluid retention, pulmonary hypertension, electrolyte imbalance, and elevated risk of edema that exacerbates COPD (Valli et al. 2004). Lower intake of sodium in the DASH diet reduces airway inflammation and improves lung function (Hirayama et al. 2010).

### 4.4 Prudent Diet

The Prudent diet is an advantageous diet known as a preventive factor for chronic diseases (Snetselaar and Lauer 2003; Szostak et al. 2013). The Prudent diet is characterized by fresh vegetables and fruits, whole grains, legumes, nuts, and also low‐to‐moderate amounts of seafood and low‐fat dairy products. In this diet, red meat products and eggs are consumed in limited quantities (Snetselaar and Lauer 2003; Sukhato et al. 2020). It is rich in fiber, antioxidants, vitamins, minerals (magnesium, potassium), and omega‐3 fatty acids. Also, lower amounts of saturated fats make this diet healthier (Bell et al. 1990; Simpson‐Yap et al. 2021).

Advantageous diets like the Prudent diet have improved lung function (Figure 2 and Table 1). Varraso, Fung, Hu, et al. (2007), in their study on 42,917 men, found that adherence to a Prudent diet was significantly related to the reduced risk of newly diagnosed COPD. Also, another study on 72,043 women, indicated a similar result. There was a significant negative correlation between the Prudent diet and the risk of newly diagnosed COPD (Varraso, Fung, Barr, et al. 2007). Analyzing 2942 adults, Shaheen et al. reported the relationship between adherence to Prudent dietary patterns on COPD and lung function. They found that high adherence to a Prudent dietary pattern is associated with a decreased prevalence of COPD in males. Also, this diet was significantly related to higher FVC in both sexes and FEV_1_ in males (Shaheen et al. 2010). Steinemann et al. (2018) indicated a significant correlation between adherence to the Prudent diet and higher FEV_1_. There are also healthy plant‐based and high‐quality diets similar to the Prudent diet, which have the same relationship as the Prudent diet on COPD patients. These diets include whole grains, fruit, vegetables, nuts, legumes, and healthy fats (Dinparast et al. 2021; Varraso et al. 2023; Varraso, Chiuve, et al. 2015; Zheng et al. 2016). A prospective cohort study found that a higher diet quality score is related to a lower risk of newly diagnosed COPD. Also, after adjusting, in participants with the highest diet quality, the risk of newly diagnosed COPD was one‐third lower than in participants with the lowest diet quality (Varraso, Chiuve, et al. 2015). Dinparast et al. (2021) revealed a significant opposite association between healthy dietary patterns and depression in COPD patients. A meta‐analysis recognized the correlation between the Prudent diet and the risk of COPD. They found high adherence to the healthy/Prudent dietary pattern reduces COPD risk (Zheng et al. 2016). Analyzing 2605 COPD, Varraso et al. found a significant association between a healthful Plant‐based Diet Index (hPDI) and the risk of COPD. After adjusting, the participant with the highest hPDI score had a 46% reduction in the risk of developing COPD (Varraso et al. 2023).

Due to the large amount of fiber in a Prudent diet, Shi et al. (2022) reported that the Prudent diet decreases inflammation mediated by the effect of gut microbiome on the immune system. The mechanism that explains the interaction between dietary fiber and gut microbiota in reducing inflammation has been mentioned before. Furthermore, this diet is rich in antioxidants, vitamins, and omega‐3 that may improve COPD. These mechanisms have been mentioned before, too.

Fish consumption is more than red meat products in the Prudent diet (Snetselaar and Lauer 2003). Nevertheless, one study did not find a significant difference between a higher intake of fish as part of the Prudent diet and the risk of newly diagnosed COPD (Varraso, Barr, et al. 2015). In addition, a high consumption of red meat products has a negative effect on COPD and is limited in the Prudent diet (Snetselaar and Lauer 2003). This mechanism is explained above. So, a lower intake of red meat in the Prudent diet is another advantage of this diet.

### 4.5 Western Diet

The Western diet is an unhealthy and modern diet characterized by high consumption of red and processed meats, unhealthy fats, high‐fat dairy products, desserts, sweets, sugary drinks, and refined grains, and also low intake of fruits, vegetables, whole grains, nuts, and fish (Carrera‐Bastos et al. 2011; García‐Montero et al. 2021). This diet is deficient in nutrients such as vitamins, antioxidants, fiber, and omega‐3. It also contains excessive amounts of omega‐6 fatty acids, SFAs, and sodium (García‐Montero et al. 2021; Rakhra et al. 2020). It increases the risk of obesity, cancer, type‐2 diabetes, dyslipidemia, cardiovascular diseases, autoimmune diseases, and cognitive disorders (López‐Taboada, González‐Pardo, and Conejo 2020; Manzel et al. 2014; Newsome, Yang, and Jobin 2023; Więckowska‐Gacek et al. 2021). Also, the Western diet can affect lung function (Brigham et al. 2015) and should be considered for COPD patients.

Unhealthy diets like the Western diet may increase the progression of COPD (Figure 2 and Table 1). Analyzing 72,043 women, Varraso, Fung, Barr, et al. (2007) reported a significant positive correlation between high adherence to the Western diet and increased risk of newly diagnosed COPD. Also, it was found that men who adhered more closely to the Western diet had a higher risk of newly diagnosed COPD (Varraso, Fung, Hu, et al. 2007). Sorli‐Aguilar et al. (2016), in a cross‐sectional study of 207 smokers without respiratory disease, showed a significant relationship between adherence to a Westernized diet and impaired lung function in women. Furthermore, Western‐liked diets have the same association with COPD patients. McKeever et al. (2010) demonstrated that a traditional dietary pattern including higher consumption of red and processed meat, added fat, potato, boiled vegetables, beer, and coffee, and lower consumption of breakfast cereal, tea, low‐fat dairy products, soy products, brown rice, pizza, fruit, and juice is related to a lower FEV_1_ and higher prevalence of COPD. A meta‐analysis showed a significant correlation between unhealthy/Western diet and COPD risk. They found that high adherence to the Western diet elevated the risk of COPD (Zheng et al. 2016).

Insufficient intake of antioxidants like polyphenols, β‐carotene, vitamin C, and vitamin E due to lower consumption of vegetables and fruit, increases oxidative stress and inflammation in the respiratory system (Heefner et al. 2024). Lower intake of fiber increases the risk of gut microbiota dysbiosis and leads to inflammation in the body and exacerbates COPD (Chassaing, Vijay‐Kumar, and Gewirtz 2017; Vaughan et al. 2019).

Red and processed meat are rich in the Western diet (Carrera‐Bastos et al. 2011). Increased meat intake is positively related to the risk of COPD (Orozco‐Levi et al. 2021). De Batlle et al. (2012), in their study on 274 COPD patients, revealed that a high intake of cured meats increases the risk of readmission. Also, a systematic review and meta‐analysis analyzed 8338 COPD patients and reported that consuming processed red meat is significantly related to the risk of COPD (Salari‐Moghaddam et al. 2019). Another study found a positive correlation between subjects with a high intake of processed meat and the risk of COPD compared to the subjects who had rarely eaten processed meat (Varraso et al. 2023). The mechanism of the effect of red processed meat is due to high amounts of nitrates, nitrites, and nitrosamine (Hord, Tang, and Bryan 2009). These compounds can produce oxidizing reactive nitrogen species and increase inflammation (Kaluza et al. 2016; Magallón, Navarro‐García, and Dasí 2019; Radi 2004). It can impair lung function and play a vital role in the pathogenesis of COPD (Barnes 2020). Furthermore, both cured meat and processed meat have high amounts of sodium (Smit 2001). In COPD patients, high sodium intake can lead to water retention due to impaired fluid processing through blood circulation and causes inflammation in the airways (Chrysohoou et al. 2022; de Leeuw and Dees 2003).

The Western diet contains omega‐6 fatty acids and saturated fats (García‐Montero et al. 2021). Inflammation plays a vital role in COPD. Red meat is rich in omega‐6, and the anti‐inflammatory properties of omega‐6 can be considered (Patel et al. 2004). The Western diet increases inflammation through epigenetic pathways, too (Marín‐Hinojosa et al. 2021). In a meta‐analysis study, it has been reported that every 50 g of processed red meat elevates the risk of COPD by 8% (Salari‐Moghaddam et al. 2019). Omega‐6 has anti‐inflammatory compounds, including lipoxins as well as pro‐inflammatory compounds including cysteinyl leukotrienes, thromboxane, and prostaglandins (Innes and Calder 2018; Patterson et al. 2012). In susceptible populations, the pro‐inflammatory effect of omega‐6 is more dominant. Also, SFAs produce an inflammatory response by activating toll‐like receptor 4 in the hypothalamus and triggering intracellular signaling networks (Milanski et al. 2009). Jiménez‐Cepeda et al. (2019) reported that higher consumption of SFAs and trans fats reduces the FEV_1_/FVC ratio.

Intake of sweets, desserts, and sugary drinks in the Western diet is high (Carrera‐Bastos et al. 2011). Min, Huh, and Moon (2020), in a cross‐sectional study of 15,961 adults, revealed that high consumption of soda decreased the mean FEV_1_/FVC ratio. Consuming excessive amounts of sugar and sugary beverages elevates the sensitivity of the allergic airway inflammatory response (Shi et al. 2012). The susceptibility of airway inflammation increases because sugar damages the immune defense system of surfactant protein D (SP‐D) (Kierstein et al. 2008). The SP‐D is a molecule in charge of the innate immune system of the lungs that interacts with cellular components and decreases the sensitivity of airway inflammatory disease (Sano and Kuroki 2005). Also, lung inflammation increases the glucose diffused from the blood into the sputum. High glucose concentrations in the sputum of COPD patients may increase the risk of bacterial infections (Garnett, Baker, and Baines 2012; Mallia et al. 2018).

### 4.6 Ramadan Intermittent Fasting Diet

Ramadan Intermittent Fasting (RIF) includes a series of specific changes in lifestyle and diet. It also includes changes in sleep and physical activity. This diet contains at least one light meal before sunrise and one large meal after sunset, and during this time between sunrise and sunset, all liquid and solid foods are avoided (Barkia et al. 2011; Mo'ez Al‐Islam et al. 2019; Ramadan 2002). In addition to the restricting food and liquids, the fasting diet in Ramadan has a series of other restrictions, such as smoking and taking oral and injectable drugs (Bragazzi 2015). Besides the amount of food, fasting can affect the quality and also the consumption of liquids (Barkia et al. 2011). The duration of fasting is between 12 and 14 h, but in some areas, it can be up to 18 and sometimes up to 22 h (Haouari et al. 2008).

Restricting food and liquids in the RIF diet may have adverse effects on COPD patients (Figure 2 and Table 1). In a pilot study on 15 fasting COPD patients, hematological indices were analyzed. After Ramadan, they found RIF can reduce white blood cells (WBC), red blood cells (RBC), hematocrit (Ht), and hemoglobin (Hb) compared to the Before‐Ramadan among hematological indices. Therefore, RIF has a negative effect on the hematological indices of COPD patients. In contrast, RIF has modified maximal mid‐expiratory flow significantly among COPD patients (Rejeb et al. 2018). Another study on 16 COPD patients showed that spirometry data were not influenced by RIF significantly in 2016 (Zouari et al. 2018). Furthermore, Mrad et al. studied 15 patients with COPD who participated in their studies. The patients had about 71 years and they fasted during Ramadan in 2017. There was no significant association between RIF and oxidant stress biomarkers, such as homocysteine, thiobarbituric acid reactive substances, and antioxidant stress biomarkers, such as ceruloplasmin, catalase, superoxide dismutase, albumin, and zinc. Also, RIF had no significant effect on the number of high oxidant stress and low antioxidant stress status (Mrad et al. 2019).

Three mechanisms of RIF on hematological factors could be included. The hydration status of the patients decreased significantly due to fasting, and then the water consumption increased (Oppliger and Bartok 2002). Another mechanism is the increase of a series of data, such as cholesterol, due to how the food is served. The series of serum data increased because a large meal is consumed instead of several small meals (Fabry et al. 1964; Irwin and Feeley 1967). These changes can affect hematological factors, such as RBC, Ht, and Hb. In addition, another mechanism has been proposed that refers to the type of food consumed. This type of diet increases the desire to consume carbohydrates and fats (Maughan et al. 2008; Sedaghat et al. 2017). As a result, this diet style reduces the intake of iron, folic acid, pyridoxine, and vitamin B12 and also increases the number of patients with anemia (Anderson and Frazer 2017; Born, Elmadfa, and Schmahl 1979; DeLoughery 2017; Lall et al. 1999). Furthermore, other nutritional deficiencies especially antioxidant deficiencies may happen during this diet and increase oxidative stress and inflammation in the lungs and exacerbate COPD (AlZunaidy et al. 2023; Heefner et al. 2024). There is no exact mechanism for spirometry changes. However, it has been hypothesized that there may be a physiological effect on the retention of spirometry data during RIF due to the lack of changes in hydration and insignificant changes in the weight of COPD patients (Zouari et al. 2018).

### 4.7 Ketogenic and Low‐Carbohydrate Diet

Low‐carbohydrate (low‐carb) diets mention dietary patterns with < 45% carbohydrate restriction. These diets increase protein and/or lipids intake to meet caloric needs (Markantes, Tsichlia, and Georgopoulos 2022; Oh, Gilani, and Uppaluri 2023). Most fruits, starchy vegetables, legumes, grains, cereals, and dairy are restricted to the low‐carb diet. These foods are replaced with foods containing protein and fat, such as cheese, eggs, meats, oils, cream, and butter (Naude et al. 2022). The Ketogenic diet, which is low in carbohydrates, provides adequate protein and high‐fat content. This diet was initially used for the treatment of epilepsy. (Masood, Annamaraju, and Uppaluri 2022). Low‐carb diets like Ketogenic diet may have positive effects on lung function and should be assessed in COPD patients (Kong and Wu 2022; Patikorn et al. 2023).

Studies showed that the Ketogenic and low‐carbohydrate diet may improve COPD (Figure 2 and Table 1). Kim, Choi, and Kim (2020) found that adequate intake of carbohydrates and protein was related to reduced COPD severity in older men and women. Also, Malmir et al. assessed the relationship between the low‐carb diet and COPD. They evaluated diet macronutrients and found a significant inverse correlation between adherence to the low‐carb diet and the odds of COPD (Malmir et al. 2021). Analyzing 60 COPD patients with low body weight, Cai et al. reported the effect of a low‐carb diet on pulmonary function. Patients were divided into two groups. The experimental group received a high‐fat, low‐carb oral supplement (28.2% CHO, 55.1% fat, and 16.7% protein) as a part of the diet, and the control group received a high‐carbohydrate diet (60%–70% CHO, 20%–30% fat, and 15% protein). Both groups had similar total energy intake. After 3 weeks, PaCO_2_ reduced significantly in both groups compared to the baseline, but PaO_2_ was significantly higher in the experimental group only. Also, FEV_1_ was increased significantly only in the experimental group. Moreover, the minute ventilation, carbon dioxide production, oxygen consumption, and respiratory quotient (RQ) were reduced significantly in the experimental group compared to the control group (Cai et al. 2003). Furthermore, Angelillo et al., in a 15‐day randomized clinical trial on 14 ambulatory COPD patients, evaluated the effect of a low‐carb diet on COPD. They divided patients into three groups, low‐carb intake (28% carbohydrate, 55% fat, and 16.7% protein), moderate‐carbohydrate intake (53% carbohydrate, 30% fat, and 16.7% protein), and high‐carbohydrate intake (74% carbohydrate, 9.4% fat, and 16.7% protein). Patients had significant weight loss after the study. In low‐ and moderate‐carbohydrate diets, the volume of carbon dioxide production was significantly lower compared to a high‐carbohydrate diet. Also, RQ and PaCO_2_ were decreased significantly in a low‐carb diet. Furthermore, FEV_1_ and FVC were significantly higher in low‐carb diet (Angelillo et al. 1985). On the other hand, A case report study of a 54‐year‐old man with COPD revealed some improvement in the disease after adhering to the ketogenic diet (70% of calories from fat). The inflammatory factors of the patient, such as granulocyte‐macrophage colony‐stimulating factor, TNF‐α, IL‐1β, IL‐6, IL‐8, and CRP, reduced into the normal range. Also, FEV_1_ increased meaningfully (37.5% increase compared to the pre‐ketogenic diet) (Norwitz et al. 2021).

The association between carbohydrate intake and respiration has been noted in several studies (Covelli et al. 1981; Efthimiou et al. 1992). Carbohydrates have a higher RQ (1.0) compared to proteins (0.8) and fats (0.7). A high‐carb diet increases the production of RQ and CO_2_, leading to worsening respiratory acidosis (Covelli et al. 1981; Patel, Kerndt, and Bhardwaj 2023). Also, in a high‐fat, low‐carb diet, consuming high amounts of fat produces ketone bodies at the amount higher than CO_2_. Nucleotide‐binding oligomerization domain‐like receptor 3 (NLRP3) suppression is associated with high production of ketone bodies. NLRP3 is a main inflammatory stimulator in COPD (Yang et al. 2015; Youm et al. 2015). Another mechanism might be related to gut microbiota. A positive change in the gut microbiome of COPD patients with low‐carb diets like the Ketogenic diet can protect against the activation of inflammatory mediators like T‐helper 17 cells. This diet reduced the abundance of *Firmicutes* and increased the abundance of *Bacteroidetes* (Ang et al. 2020; Olson et al. 2018). Therefore, lung function and respiratory condition improve by adhering to a low‐carb diet.

### 4.8 High‐Protein Diet

Proteins are a common source of energy that contains more than 18% of the total daily energy intake in High‐protein diets (Bortolotti 2010). A High‐protein diet contains animal origin and vegetable proteins. Food with animal origin are dairy products, meats, poultry, eggs, and seafood. Vegetable proteins includes soy protein, nuts, seeds, legumes, and tofu (González‐Pérez and Arellano 2009; Lim et al. 2021; Marcus 2013). If fat intake from animal sources is not controlled, having a High‐protein diet for a long time increases the risk of cardiovascular diseases (Hu et al. 1997). Decreased protein intake through diet and protein synthesis and breakdown imbalance leads to protein reduction in COPD patients (Agusti et al. 2003; Mallampalli 2004).

Some studies found a positive relationship between lung function of COPD patients and adhering to a High‐protein diet (Figure 2 and Table 1). Yazdanpanah et al. assessed 63 patients with COPD. According to the GOLD stages, the participants were divided into three groups. They found that the requirements for energy and protein are more than their intake for each group. Also, they indicated a significant positive relationship between the amount of protein consumption and FVC and vital capacity (Yazdanpanah et al. 2010). Analyzing 13 patients with severe COPD in a randomized clinical trial study, Møgelberg et al. (2022) reported that a high‐protein diet with physical activity improves peripheral muscle function.

Chronic and progressive dyspnea of COPD patients makes them choose a sedentary lifestyle to prevent exertional dyspnea. It results in the deconditioning of skeletal muscle (Miravitlles et al. 2014; O'Donnell et al. 2020). Therefore, energy and protein demand are higher in patients with COPD, and insufficient intake leads to respiratory muscle weakness (Akner and Cederholm 2001; Schols 2002). Refeeding helps to strengthen the respiratory muscles of patients (Pingleton 1996). In this regard, some studies reported that protein supplementation in COPD patients improves inflammation, muscle strength, quality of life, and exercise tolerance. (Ahmadi et al. 2020; Sugawara et al. 2012). On the other hand, it is advisable to control the consumption of animal‐origin proteins due to their potential to induce inflammation (Wang et al. 2022).

## 5 Conclusion

This review summarized recent publications on the association between healthy and unhealthy dietary patterns and the prevalence and development of COPD. According to the current literature, it could be concluded that some dietary patterns, such as Mediterranean, DASH, Prudent, Ketogenic, and High‐protein diet, play a vital role in the prevention and treatment of COPD. On the other hand, Western and RIF diets may increase the prevalence and progression of COPD. Although our understanding of the molecular mechanism behind the observed dietary effects is still limited, it appears that foods and nutrients may have antagonistic and synergistic interactions in COPD. Therefore, using nutritional recommendations could be beneficial in the prevention and treatment of this disease.

## Author Contributions

**Mohammad Vahedi Fard:** conceptualization, investigation and data curation, writing – original draft. **Kimia Mohammadhasani:** investigation and data curation, methodology, writing – original draft. **Zahra Dehnavi:** writing – review and editing, creating visual representations. **Zahra Khorasanchi:** project administration, writing – review and editing.

## Conflicts of Interest

The authors declare no conflicts of interest.

## Data Availability

Data sharing does not apply to this article as no datasets were generated or analyzed.
